# Improving the SSIR Method: Implementation of an Exhaustive Multilevel Scan for Categorical Variables

**DOI:** 10.3390/ijms27041972

**Published:** 2026-02-19

**Authors:** Emili Besalú

**Affiliations:** Institut de Química Computacional i Catàlisi (IQCC) and Departament de Química, Universitat de Girona, 17071 Girona, Spain; emili.besalu@udg.edu

**Keywords:** SSIR method, SSIR with multilevels, anti-HIV compounds, analogue series, ranking, structure–activity relationships, in silico synthesis

## Abstract

The Superposing Significant Interaction Rules (SSIR) method is briefly revisited. The original version of the algorithm consists of dealing with rules that evaluate the individual presence or absence of substituents in the molecular structures of a combinatorial chemical family. Here, an extension is developed, allowing for the systematic consideration of multilevel conditions, i.e., the presence or absence of not only single residues but also groups of residues attached to a particular substitution site. This possibility expands the universe of possible definable rules. Then, the original SSIR version becomes a particular case of the framework presented in this manuscript. A numerical example involving a family of anti-HIV compounds is provided to illustrate the application and potential of the extended method. Procedures for generating predictions for new in silico virtual molecular structures are also shown.

## 1. Introduction

It is well known that the procedures of automated learning have a large impact on several fields, such as physics, chemistry [1,2], or even economics, among many others. In particular, many applications and variants have been developed to conduct studies in materials science, pharmacology, cheminformatics, and molecular design [3]. Within the related field of structure–activity relationships (SAR), the Superposing Significant Interaction Rules (SSIR) method [4] was developed as an automated procedure that is able to generate new optimal molecular candidates in a combinatorial fashion. The procedure was initially tested for constructing a consensus model aimed at predicting binding affinities [5]. Since then, several publications have demonstrated its application in various areas of molecular modeling and related fields (see, for example, reference [4] and the citations therein).

The SSIR method is useful for qualitatively ranking a series of compounds only using very simple symbolic codifications of molecular descriptors. These descriptors can be encoded symbols representing the possible substitution residues available at the distinct molecular sites of a common molecular scaffold. The method generates a set of statistically significant rules that collectively form an expert voting system. The voting procedure generates molecular rankings that are expected to correlate with a dichotomized version of the property under investigation. The significant rules are selected according to a simple and straightforward procedure based on a statistical parameter (a *p*-value) once the molecular set under study has been partitioned into two groups: the subset of interest (e.g., active, non-toxic, exhibiting a desirable property, etc.) and the complementary subset of compounds of non-interest.

As mentioned, the symbolic codification of a combinatorial family of molecules is derived from the labeling of the distinct residues placeable at the different substitution sites of the common molecular scaffold (however, alternative descriptor categorizations are also possible, but they are not described here). The original version of SSIR deals with basic rules that state the presence or absence of particular residues in particular molecular sites. The experiences lead to thinking in terms of dealing with the extension of the rules’ definition, accounting for the presence or absence of groups of residues. This article focuses on this aspect. First, the main features of the classical SSIR procedure are presented using a toy example. Then, the extension of the rule definition (the multilevel approach) is developed. It follows the practical application to an anti-HIV set of compounds taken from the literature. Both SSIR procedures are compared with the methodology used in the source of information. Finally, it is shown how easily the SSIR method produces predictions for new molecular candidates.

## 2. Materials and Methods

### 2.1. A Toy Application Example and SSIR Rules

**Figure 1:**
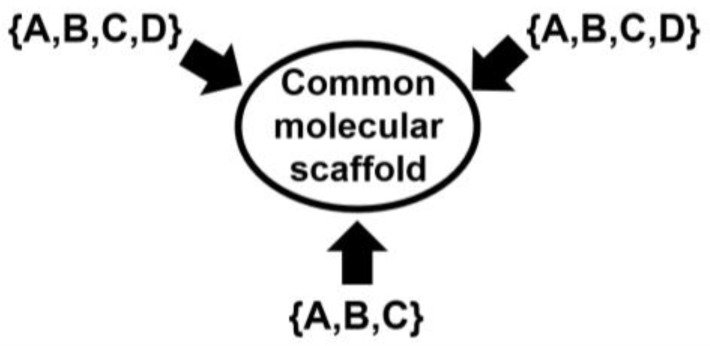
Representation of the combinatorial space expanding the toy molecular set {A,B,C,D}⊗{A,B,C,D}⊗{A,B,C} derivable from the common scaffold presenting three substitution sites. Each substitution site admits several residues, which are represented by letters. See text for details.

A simple artificial illustrative example is provided here to highlight the main features of the SSIR method. This example uses a small toy dataset from a family of combinatorial congeneric compounds. The common molecular scaffold has three substitution sites (see Figure 1): The first and second allow four possible residues {A,B,C,D}, while the third permits three: {A,B,C}. As explained in previous publications, the same letter (e.g., A) may represent different chemical substituents depending on the substitution site. The full combinatorial molecular library can be denoted by {A,B,C,D}⊗{A,B,C,D}⊗{A,B,C}.

Each compound in the molecular family is represented as an ordered triplet of characters. For example, a compound may be denoted as ACB (i.e., the molecule exhibits residue A at site 1, residue C at site 2, and substituent B at the third site of Figure 1). In total, the full combinatorial space includes 4 × 4 × 3 = 48 compounds. Table 1 presents 9 molecules from this family for which a target property is known. Each molecule is labeled as either of interest (Y) or of non-interest (N) based on the property outcome.

Can the reader identify a simple rule that captures all or most of the compounds of interest? As a first approximation, one may suggest a rule that selects compounds containing residue A at the second substitution site. This corresponds to the rule pattern X[A]X (where X denotes a wildcard, i.e., any residue), which matches four compounds in Table 1 (AAA, AAB, AAC, and CAA), three of which are labeled as of interest. This is an example of a first-order rule, as it focuses on a single substitution site while allowing any residue at the first and third sites. In this case, it is said that the rule embraces or matches four compounds. Once a rule is defined, it is statistically evaluated according to the number of molecules of interest the rule embraces. For instance, the rule X[A]X encompasses the four cited molecules from Table 1, three of which are of interest. The statistical significance of this rule is assessed by calculating the probability that, in a sample of 9 compounds (i.e., the set of Table 1), a randomly selected subset of 4 (the ones selected by the rule) would contain 3 or more compounds of interest. This probability is derived from the hypergeometric term (see below)

*p*(3+,4;3,9) = *P*(3,4;3,9) = *C*(3,3)·*C*(6,1)/*C*(9,4) = 1/21 = 4.8%

where *C*(*m*,*n*) denotes the combinatorial number of *m* elements taken in groups of *n*. Since this probability is smaller than a predefined *p*-value threshold (say 5% in our example), the rule X[A]X is included in the voting system. The voting system collects all the definable significant rules, and it acts as an expert agent at the test or prediction stages in the following way: Any new virtual compound is confronted with the significant rules, and for each rule that matches with it, the molecule receives a vote. The vote can be positive or negative, as described below and in the references.

The classical SSIR method can deal with a variety of rule orders. For instance, all possible first-order rules can be generated, such as [A]XX, [B]XX, …, X[A]X, X[B]X, …, XX[C], and a total of 11 such rules are definable in the example. Also, second-order rules can be considered, such as [A][A]X, [A][B]X, …, X[D][C], resulting in 40 combinations. In this example, any of the 48 possible third-order rules (e.g., [A][A][A], [A][A][B], …, [D][D][C]) provides no additional insight, as it corresponds to a single compound and thus lacks generality.

As shown above, each rule is statistically evaluated. In general, the hypergeometric probability accounts for the likelihood that, in a library of *L* items (where *I* of them are of interest), exactly *i* items of interest are found when randomly selecting *s* compounds. This probability is given by the following term:

*P*(*i*,*s*;*I*,*L*) = *C*(*I*,*i*)·*C*(*L* − *I*, *s* − *i*)/*C*(*L*,*s*).

The significance (*p*-value) of picking up *i* or more items of interest is obtained by adding the corresponding values for *i*, *i* + 1, *i* + 2,…, min(*I*,*s*) [4]:

*p*(*i*+,*s*;*I*,*L*) = *P*(*i*,*s*;*I*,*L*) + *P*(*i* + 1,*s*;*I*,*L*) + … + *P*(min(*I*,*s*),*s*;*I*,*L*).

The reader may check that the most significant rule of order one definable in the proposed example is [A]XX. This rule embraces the first three compounds in the dataset, all of which are of interest. Its associated *p*-value is *p*(3+,3;3,9) = *P*(3,3;3,9) = 1.2%, and this feature allows the rule to belong to the expert voting system. Other rules are not sufficiently significant to be included in the voting system because their associated probabilities exceed the predefined *p*-value threshold of 5%. For example, the rule XX[A] matches three compounds in the library (AAA, BDA, and CAA), but only one of them is of interest. The statistical significance of this rule is only

*p*(1+,3;3,9) = *P*(1,3;3,9) + *P*(2,3;3,9) + *P*(3,3;3,9) = 76.2%

Rules can account not only for the presence of a specific residue but also for its absence. In such cases, negation operators are used [4]. When negation terms are included, the total number of possible rules increases. For instance, the rule X[|A]X defines the subset of molecules that do not contain residue A at the second substitution site. In our example, this rule covers five compounds, none of which are labeled as of interest. The statistical significance of this rule, therefore, corresponds to the trivial probability of randomly selecting five compounds and obtaining zero or more items of interest:

*p*(0+,5;3,9) = *P*(0,5;3,9) + *P*(1,5;3,9) + *P*(2,5;3,9) + *P*(3,5;3,9) = 100%

This rule is not significant at all in the sense described above, but its information is also useful: It constitutes a rule attached to a low probability of collecting compounds of interest. In this case, these kinds of rules can also be included in the voting system but are attached to a negative vote, i.e., each molecule that fulfills the rule will receive a negative vote instead of a positive one. All in all, the significant rules that are included in the voting system are:

- The rules attached to a positive vote: the ones for which its *p*-value is less than the *p*-value threshold.
- The rules attached to a negative vote: the ones for which the quantity of 100—*p*-value is less than the *p*-value threshold.

Once all significant rules have been collected, the resulting set of rules constitutes and functions as an expert system: When a new compound is evaluated, each matching rule contributes a vote (positive or negative). So, any new molecular candidates will collect the sum of votes given by the rules that embrace them and belong to the expert system. The more positive votes a new compound receives from the voting system, the higher the probability that the molecule will be of interest.

### 2.2. SSIR Procedure

**Figure 2:**
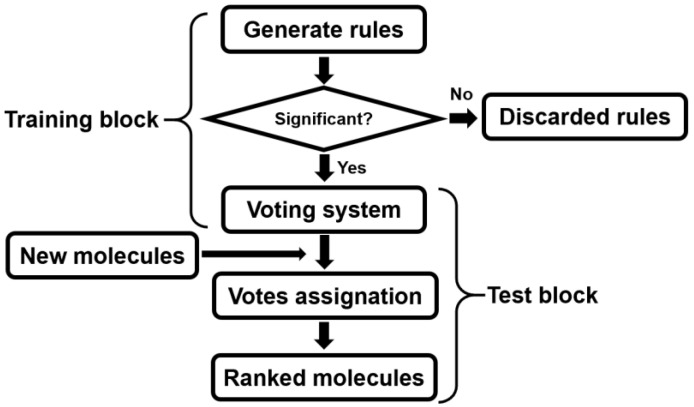
Schematic flow chart followed by the SSIR method. In the training block, the rules are generated and selected from the training set. The significant rules constitute the the expert voting system. Afterwards, this voting agent is used in the test block in order to evaluate new external molecules. Each new molecule receives a sum of votes. These sums are used to rank the molecules.

Here, the general procedure followed when the SSIR method is applied is briefly presented. Conceptually, the procedure is the same as the one followed by the SAR protocols (training and constructing a model; applying the model to other external compounds). The flow chart is depicted in Figure 2, and it consists of the following steps.

Once the training molecular set is prepared and the site residues are defined, all the rules of a certain range of orders (decided by the user) are generated. Then, each rule is evaluated in terms of significance (as explained above), and only those declared as significant (according to the predefined *p*-value threshold) are kept and included in the expert voting system. This block of actions constitutes the training procedure. At the end of the training procedure, the expert system collects a set of selected rules, each one attached to a positive or a negative vote. These rules are ready to act on new compounds.

Then, new molecules (usually a test set for which a prediction has to be obtained) are evaluated against each rule of the voting system. Each compound is evaluated against each of the selected rules, and in the case of a match, the compound receives a new additive vote (positive or negative according to the rule classification). Along this test block, each new compound receives a final score. This score is the sum of the votes assigned by the significant rules that match (cover) the compound. This score serves to establish a ranking of the compounds. It is expected that the scores correlate with the probability that a compound is of interest. If the binary property (the quality to be or not to be of interest) is known for the sorted set of molecules, then the result can be parametrized *a posteriori* with standard classification parameters (see below).

### 2.3. Defining New Rules from the Multilevel Approach

The main objective of this article is to present a generalization of how the rules are defined. As shown, a classical rule involves a single specific residue at each site, as in the rule X[A]X. An extension of the rule is found when a negation term is introduced. For instance, the rule X[|A]X represents the case where any residue except A is allowed at the second substitution set. This exemplifies a particular multilevel case, i.e., a rule that provides the possibility to choose among several residues at a fixed site. This rule can be written in an equivalent multilevel form as X[B,C,D]X. In general, any negation can be expressed using the proper multilevel notation. For example, the negation term [|A] is equivalent to [B,C,D], or the negation [|(B,C)] = [|B,|C] is the same as [A,D], or the rule X[|B,C]X is the same as X[A,C,D]X.

The possibility of generating many multilevel combinations opens the door to considering new and more complex rules.

#### 2.3.1. Multilevel Terms

Let us consider again the same molecular library presented in the previous subsection: {A,B,C,D}⊗{A,B,C,D}⊗{A,B,C}. For the first substitution site, 14 non-trivial multilevel factors or terms can be defined in order to enter into a rule:

- Four terms of order 1: [A], [B], [C], and [D], meaning that only residue A is accepted, or only residue B, C, or D. These terms are equivalent to the ones appearing above in Section 2.1 (based on the classical SSIR approach).
- Six terms of order 2: [A,B], [A,C], [A,D], [B,C], [B,D], and [C,D], meaning that residue A or B is accepted or residue A or C, and so on. These are examples of multilevel terms, i.e., choices of subsets of residues where two or more of them can be selected.
- Four terms of order 3: [A,B,C], [A,B,D], [A,C,D], and [B,C,D]. In this case, each one of these multilevel terms is equivalent to a negation term. For instance, the first one is the same as [|D].

Similar terms of distinct orders can be defined for the second and third sites. Then, a multilevel rule can be defined by combining the terms (one for each site). For instance, the rule [B][A,C,D][A,B] is of order 3 because it focuses on three sites. The rule is defined from a first term of order 1, a second term of order 3, and a third term of order 2. The rule points to the compounds presenting fixed residue B at site 1; residues A, C, or D at site 2; and, at the same time, residues A or B at site 3. Examples of this kind of covered compounds are BAA, BDB, BCA, and so on. In Table 1 above, only the compound BDA is embraced by this rule. To illustrate another example, consider the following rule of order 2 involving multilevel terms of order 2 for the first and second sites (and, necessarily, the wildcard for the third site): [B,C][C,D]X. This rule encompasses any compound that has either residue B or C at the first site and, simultaneously, residue C or D at the second site, irrespective of the substitution at the third site. This rule matches the compounds BCC, BDA, and CDC in Table 1.

#### 2.3.2. Rules Composed by Multilevel Terms

The SSIR rules are obtained by juxtaposing a series of terms, each one associated with a molecular site. For instance, following the example, there are 14 rules of order 1 of the form [·]XX, i.e., involving only terms specifying characteristics of the first site. The possibilities are:

- *C*(4,1) = 4 rules for which the relevant term is of order 1: [A]XX, [B]XX, [C]XX, and [D]XX.
- *C*(4,2) = 6 rules where the term is of order 2: [A,B]XX, [A,C]XX, [A,D]XX, [B,C]XX, [B,D]XX, and [C,D]XX.
- *C*(4,3) = 4 rules involving the term of order 3: [A,B,C]XX, [A,B,D]XX, [A,C,D]XX, and [B,C,D]XX.

The same can be said for the second site. The third substitution site offers 6 possibilities for rules of the type XX[·]. In general, for a site able to accommodate *m* residues, the number of definable terms of order *r* is *C*(*m*,*r*), and the total number of relevant terms attached to the site is obtained by covering the term order from 1 up to *m* − 1:

*C*(*m*,1) + *C*(*m*,2) + … + *C*(*m*,*m* − 1) = 2*^m^* − 2.

This gives the number of rules of order 1 that can be generated from the site. So, there are 2^4^ − 2 = 14 rules of order 1 of the kind [·]XX, also 14 rules of order 1 of the kind X[·]X, and 2^3^ − 2 = 6 rules of order 1 of the kind XX[·] involving site 3.

There are three kinds of rules of order 2: [·][·]X, [·]X[·], and X[·][·]. The first kind allows for 14 × 14 = 196 multilevel combinations, while the second and third kinds generate 14 × 6 = 84 rules each. Overall, there are a total of 364 multilevel rules of order 2. Regarding the rules of order 3, i.e., according to the pattern [·][·][·], a total of 14 × 14 × 6 = 1176 combinations can be generated.

In this context, there are two trivial terms worth mentioning:

- The void or empty term (of order 0), denoted as [], represents the choice of placing no residue at the corresponding substitution site. This is not valid chemically, as it does not correspond to a feasible compound.
- Conversely, the full term [A,B,C,D] represents the presence of any residue at the first or second substitution sites. This is equivalent to the wildcard symbol X.

This explains the significance of the subtraction of 2 units in the above formula. The value 2*^m^* − 2 corresponds to all the possible subsets that can be formed with the *m* residues (2*^m^*) but excluding the void and full terms. This convention ensures that the site retains some (but not all) of the possible residues, thereby preserving the order of the rule in which the term participates. In other words, when rules of a fixed order are being systematically generated, the inclusion of additional wildcard X terms must be avoided, as their presence reduces the effective order of the rule by one unit. For example, when generating second-order rules with multilevel terms of the form [·][·]X, the rule [ABCD][BC]X must be avoided because it is equivalent to X[BC]X, which is actually a first-order rule.

Overall, the total number of rules (of any order distinct from 0) that can be constructed from a molecular scaffold with *s* substitution sites, where each one accommodates *m*_1_, *m*_2_, …, *m_s_* residues is given by

$$R=\left[\prod_{i=1}^{s}\left(2^{m_{i}}-1\right)\right]-1$$

where each term of the form (2*^m^* − 1) accounts for all possible combinations of residues at a given substitution site, excluding the empty set term but including the full set of residues (i.e., the wildcard X). As noted above, whenever a wildcard is included, the order of the rule decreases by one unit. In the previous formula, the final term subtracting one unit accounts for the exclusion of the rule of order zero XX…X, a trivial and useless case that embraces all the library compounds. In our specific example, the total number of relevant rules that can be generated is

(2^4^ − 1)·(2^4^ − 1)·(2^3^ − 1) − 1 = 15 × 15 × 7 − 1 = 1574.

It must be noted that the rules that involve multilevel terms inherently include those arising from negation terms. As said above, any negation term can be equivalently expressed as a multilevel factor not involving the negation notation.

## 3. Application Example: Anti-HIV Nucleoside Compounds

Some years ago, Estrada et al. [6] investigated the activity of a set of anti-HIV nucleoside compounds using TOPS-MODE and 2D/3D Connectivity Index methodologies. In the present work, the set of molecules is retrieved and recoded following the SSIR approach. The binary activity property (active/inactive against HIV) is modeled using two strategies: first, by the original SSIR method without multilevels; and secondly, by the new proposed extension incorporating multilevel conditions. Table 2 presents the codification of residues used in this study. Each letter stands for a specific residue usable as a molecular fragment in a particular substitution site. So, the same letter or symbol can be used for distinct residues provided that these substituents are fragments to be placed at distinct scaffold sites. It has to be pointed out that certain pairs of sites from the original publication (V-R_1_, W-R_2_, X-R_3_, and Y-R_4_) have been encoded in a fused manner. This is possible due to an interesting property of SSIR: it operates entirely on symbolic codifications of residues, allowing such structural flexibility when possible.

The molecular dataset described in Table 3 of the original publication presents 133 compounds. However, compounds numbered 70 and 131 are duplicates. Here, the compound 131 has been removed, resulting in a final database of 132 unique molecules. The training subset includes 61 compounds, as labelled in the original reference. This set is referred to here as the training set or simply the TRN set. The set labeled as “cross-validation” in Ref. [6] (molecules labeled with letter a in the numbering of the original Table 3) consists of 56 molecules. This set here is referred to as the first external set (Ex1 set). Similarly, the original “external prediction set” that comprises 15 compounds (labeled with letter b in the numbering of the original Table 3) is referred to here as the second external set (Ex2 set). Table 3 lists the entire molecular family of 132 compounds. The compounds presented in Table 4 of the original reference are not considered in this study, as their molecular scaffold differs from that of the compounds of the original Table 3. Note that each string of letters (e.g., the first entry of Table 3 FABFBIDLJ) stands for (represents) a molecule according to the substituents defined in Table 2 along the nine ordered sites placed at the scaffold depicted in Table 3 of Estrada’s article. The total number of theoretically possible compounds is *M* = 8 × 3 × 5 × 22 × 2 × 12 × 5 × 15 × 15 = 71,280,000, provided that all the substituents can be combined without restrictions. For this molecular set, the condition of a molecule being of interest or of non-interest is determined by the value of the effective concentration reaching a 50% of maximum effect (EC_50_) on the Metallothionein4 (MT4) assay. Active compounds are those having EC_50_ ≤ 10 µM, and inactive ones are those having EC_50_ > 10 µM. The training set is composed of 21 active compounds and 40 inactive compounds. The Ex1 set has 12 active compounds and 44 inactive compounds. The Ex2 set is unbalanced, presenting only 2 compounds of interest out of a total of 15 molecules.

In the original reference, from a variety of topological, topographic, and quantum chemical descriptors, a training discriminant function was derived. This function correctly classified 85.7% of the active compounds and 81.8% of the inactive ones. This gives an overall performance (concordance or accuracy) of 83.1%. The percentage of false positives (false actives) was 6.2%, while the proportion of false negatives (false inactive) was 4.6%. The model obtained for training was subsequently applied to the Ex1 set (56 compounds), achieving 83.3% and 86.4% of correctly classified active and inactive compounds, respectively (global performance of 85.7%). The percentage of false classification for the actives was 10.7% and 3.6% for the inactives. The same model was also tested on the Ex2 set, yielding 85% correct classifications without any false negatives.

### 3.1. SSIR Results Without Multilevel Treatment

Table 4 presents the classification parameters obtained when the original SSIR procedure (without negation terms) is trained with the set of 61 TRN compounds. For instance, in the training block of rules generation (see Figure 2), a total of 56 rules of order 1 can be defined. Of these rules, only three have a *p*-value equal to or less than a prefixed threshold of 20%. This means that, in the test block, a test molecule can receive votes from three rules at most.

The resulting rules were applied to the Ex1 and Ex2 sets (see the test block of Figure 2), consisting of 56 and 15 compounds, respectively. The model performs well on the first external set: The ranked series of molecules achieved, for some cases, areas under the receiver operating characteristic curve (AU-ROC) [7,8,9] above 0.8, which indicates good predictive power. In contrast, the second external set (Ex2) could not be properly analyzed, and the predictions for this set are notably poor.

A common trend observed in many SSIR calculations is that the AU-ROC value for the training set (AU-ROC_TRN_) typically increases initially as more significant rules are incorporated into the voting system. However, this increase does not guarantee improved performance, particularly when too many rules are added. The corresponding leave-one-out (L1O) [10] cross-validation (CV) results (AU-ROC_L1O_) over the training items serve as an indicator of the model’s predictive power. The L1O procedure consists of obtaining a prediction (i.e., obtaining a score from the voting system) for each compound, one at a time. This is done by selecting a single compound and extracting it from the training set. Then, the reduced training set is used to obtain a predictive model (training block in Figure 2), and finally, the test block is executed for a single compound: the one that was originally extracted from the original training set. This allows us to assign a predictive score to the extracted compound. Then, the test compound is repositioned into the original training set. This action is repeated for all compounds, one at a time. At the end, each compound of the original training set bears a predictive score. This set of scores establishes a molecular ranking that can be evaluated by classification parameters, such as the AU-ROC.

Normally, while AU-ROC_TRN_ increases and reaches a maximum, AU-ROC_L1O_ tends to peak earlier at small *p*-values, typically when fewer rules have been included. This pattern can be observed in the third column of Table 4. The most realistic measure of predictive performance comes from testing the model on external molecular libraries that were not involved in training or L1O processes. The last two columns of Table 4 are obtained during the test block of Figure 2 and report the AU-ROC values for the two external sets, AU-ROC_Ex1_ and AU-ROC_Ex2_, respectively. One of the best-performing models based on the classical SSIR method was obtained using rules of order 3 with a significance threshold (*p*-value) set at 0.15. In this case, 18376 rules were generated, of which 96 (0.52%) were statistically significant, constituting the expert voting system. Applying this model to the Ex1 set yielded an AU-ROC_Ex1_ value of 0.853, indicating strong predictive performance. In contrast, the results for the Ex2 set were poor. This bad result can be attributed to several factors: the small size of the set (only 15 compounds), its limited overlap with the chemical space covered by the training set (too few rules match the tested compounds), and its poor statistical balance (only 2 out of the 15 compounds are active). These limitations result in AU-ROC values close to 0.5, which corresponds to a random and non-informative classifier. Despite better results for the Ex2 set being presented below, the conditions explained here point out that this external set is not suitable for assessing the predictive power of a method. The set has been considered here for comparison purposes with the original article of Estrada and coworkers.

For the Ex1 set, SSIR predictions at the optimal voting frontier (compounds having up to a score of 10 votes) yield the following classification metrics (confusion matrix):

**Classical SSIR calculation for external set Ex1**

n_Ex1_ = 56 (Positives or of interest = 12, Negatives or of non-interest = 44)

(Sensitivity 75%) True Positives = 9 | 41 = True Negatives (Specificity 93.2%)

False Positives = 3 | 3 = False Negatives

Accuracy = 89.3%, Precision = 75.0%, F1 = 75.0%, AU-ROC = 0.853

The percentage of true positives among the actual positives is referred to as sensitivity, while the corresponding metric for negatives is known as specificity (see ranking parameter definitions in [3,11]). Compared to the model proposed by Estrada and co-workers, the SSIR model yields one additional false negative (three instead of two), but achieves a better true negative rate (93.2% versus 86.4% or 41 correctly classified negatives compared to 38). The overall performance of the SSIR model is accounted for by the accuracy parameter: 50 out of 56 (89.3%) compounds were correctly classified, thereby improving on Estrada’s model, which correctly classified 48 compounds. However, the classical SSIR model failed to accurately classify the Ex2 set.

### 3.2. SSIR Results with Multilevel Treatment

With the aim of improving prediction accuracy, calculations were performed using the SSIR method, incorporating rules based on multiple levels. In this case, only multilevel terms up to order 2 were considered. Table 5 presents the corresponding results.

**Figure 3:**
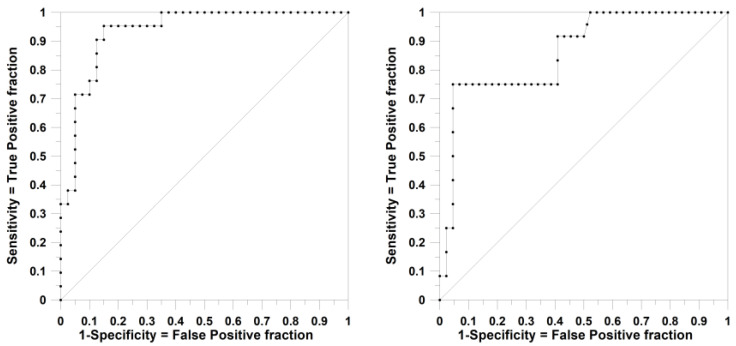
ROC curves attached to the TRN set (left, AU-ROC = 0.936) and the predictions of the Ex1 set (right, AU-ROC = 0.863) using multilevels (rules of order 3, terms of order 2, *p*-value of 0.15). See text for details.

As explained above, the multilevel approach constitutes an extension of the classical SSIR framework. It allows for the generation and evaluation of a much greater number of rules. As a consequence, the expert system incorporated more voters. This increase in the number of significant rules often correlates with an improvement in the AU-ROC value during the training process, although such improvement is not guaranteed in every case. It is expected that enhancement in the AU-ROC_TRN_ may also translate into better prediction performance. This trend is evident when comparing the results in Table 5 with those in Table 4. The total number of rules (the generated ones and the ones included in the expert system) has increased substantially, and AU-ROC values of 0.9 or higher are readily achieved during training compared to the outcomes shown in Table 4. Specifically, considering the results for rules of order 3 with a *p*-value threshold of 0.15, the predictions for the Ex1 set reach an optimal frontier point at the rank score of 3025 votes, yielding an AU-ROC of 0.863 (in Figure 3, see the corresponding ROC curves attached to training and prediction) and the following classification metrics.

**SSIR with multilevel calculation for external set Ex1**

n_Ex1_ = 56 (Positives or of interest = 12, Negatives or of non-interest = 44)

(Sensitivity 75%) True Positives = 9 | 42 = True Negatives (Specificity 95.5%)

False Positives = 2 | 3 = False Negatives

Accuracy = 91.1%, Precision = 81.8%, F1 = 66.7%, AU-ROC = 0.863

Qualitatively, the results improve by correctly identifying one additional true negative, thereby increasing both specificity and accuracy compared to the previous models of Estrada and the classical SSIR approach. The relevant point here was that the current model successfully produces accurate predictions for the Ex2 set, despite the particular ill-conditioned nature of this dataset that is explained above. The optimal point at the score of 520 votes gives the following results for this model:

**SSIR with multilevel calculation for external set Ex2**

n_Ex2_ = 15 (Positives or of interest = 2, Negatives or of non-interest = 13)

(Sensitivity 100%) True Positives = 2 | 11 = True Negatives (Specificity 84.6%)

False Positives = 2 | 0 = False Negatives

Accuracy = 86.7%, Precision = 50.0%, F1 = 66.7%, AU-ROC = 0.846

For the Ex2 set, the multilevel SSIR model slightly improves Estrada’s model (86.7% against 85% for global classifications), also without any false negatives. However, it should be noted that Estrada’s model was applied to more molecules (19 distinct molecules instead of 15). On the other hand, the present study is mainly focused on demonstrating that the inclusion of multilevel terms improves the classical SSIR methodology.

One advantage of the SSIR method, when applied to combinatorial chemistry-related fields, lies in its ability to handle molecular data symbolically. This allows for a fast preparation of the calculations, with minimal pre-processing tasks. The complexity of other methods (such as that of Estrada and coworkers considered here) is much greater than that of SSIR. For instance, by using SSIR, the codification of a set and the obtention of a model of voting can take only several minutes in some cases. On the other hand, the main disadvantage of SSIR is related to the need to use a single molecular scaffold common to all the studied databases. This requirement can be circumvented sometimes if the molecules are described by fingerprints or by continuous descriptors, which can be segmented in order to obtain factor levels. Another limitation of SSIR is that the training process should ideally include all the possible substituents that could be found later in external sets when executing the test block of Figure 2. Other methodologies allow obtaining predictions for molecules having molecular residues not present in the training compounds. If this circumstance is met when using SSIR, the method loses efficacy. This is so because the presence of a substitution found only in test compounds (but not in training) will reduce the number of rules of the expert system that can be applied to the molecule, i.e., those that match with it. In these cases, the test molecules are not well described.

### 3.3. Additional Tests for Assessing the SSIR Predictive Power

As part of the ranking parameters presented above, two CV procedures have been considered in order to measure the predictive power and stability of the multilevel SSIR methodology. A series of 100 randomized 10-fold CV calculations has been conducted. Additionally, a balanced leave-two-out (BL2O) procedure has also been performed. In all the cases, the training steps involved rules of order 3 (with term levels of order 2) and a *p*-value threshold of 0.15.

#### 3.3.1. Series of 10-Fold Cross-Validations

A 10-fold CV calculation consists of partitioning the whole training set into 10 parts as uniformly as possible. In particular, the set of 61 compounds has been divided into nine subsets of six molecules and an additional subset of seven compounds. The subsets of six molecules contained four inactive compounds and two active ones. The last subset of seven compounds contained three active molecules. All these partitions have been done randomly. Then, a series of 10 training and test calculations starts. At each step, nine of the subsets are used for training (and generation of the voting system), and the left-out subset acts as a test. In this way, after the training, the test subset receives a prediction. This procedure is repeated iteratively, ensuring that each of the 10 original subsets acts once as a test set. At the end of the procedure, each subset, and hence, each one of the compounds, received a prediction. The final result of this 10-fold CV provides classification parameters attached to this series of predictions.

**Figure 4:**
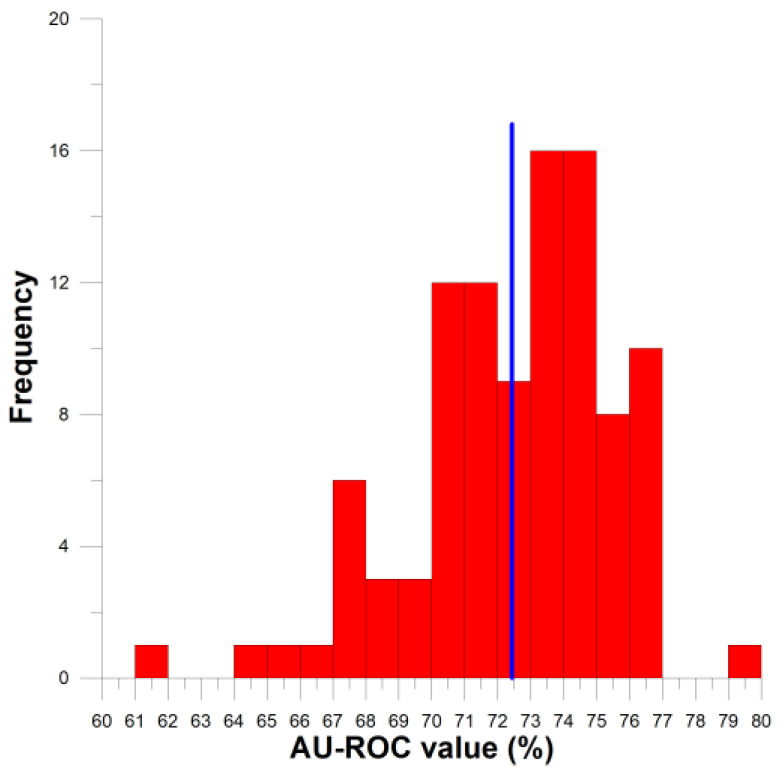
Histogram of the AU-ROC values obtained with the 100 random series of 10-fold CV calculations. The central line signals the mean value.

To avoid depending solely on a single, specific calculation, the procedure described in the previous paragraph has been repeated 100 times. All the series of 10 subset partitions were distinct as the molecular groupings were generated randomly. This procedure allowed the collection of a series of 100 classification parameters, e.g., AU-ROC values, showing a distribution. Figure 4 depicts the corresponding histogram. The distribution has a mean of 72.44 (*n* = 100, *s* = 3.02). In this way, it is shown how the method performs when predicting (in CV mode) from a statistical point of view. The tendency is that the majority (95%) of the AU-ROC values for the predictions are in the interval [0.67,0.77]. This range of values is consonant with the results obtained in Section 3.2. It is worth noting that along the cross-validation series, the training and prediction actions were performed for sets of a distinct number of elements than those of Section 3.2; this is not to say that the external sets were always distinct.

#### 3.3.2. BL2O Calculation

A BL2O calculation is a particular CV procedure that can be applied to sets where the goal property is dichotomic. In the case presented here, the training set of 61 structures contained 21 active compounds and 40 inactive ones. The BL2O procedure consists of performing a designed series of leave-two-out (L2O) procedures. In an L2O calculation, two original training compounds are left apart. The remaining molecules serve to construct the training model, which, in turn, is used to obtain a prediction for the two left-out structures. In BL2O calculations, each L2O step involves the extraction of a test set formed by one active molecule and one inactive one. This procedure is repeated for all possible pairs that can be constructed in this way. The total number of test subsets that can be formed is obtained by the number of active compounds times the number of inactive ones. In the present example, a total of 21 × 40 = 840 calculations were conducted. By doing that, each active compound received 40 predictions (one prediction for each inactive compound that was acting as a companion forming the left-out test subset). Similarly, each inactive compound received a total of 21 predictions. All the test votes were collected, but in order to balance the results, the votes received by the active compounds were multiplied by 21, and the votes originally received by each inactive compound were multiplied by 40. This series of uniformized (balanced) votes provided the following ranking parameters:

**SSIR with multilevel calculation for the training set (BL2O-CV procedure)**

n_BL2O_ = 61 (Positives or of interest = 21, Negatives or of non-interest = 40)

(Sensitivity 95.2%) True Positives = 20 | 18 = True Negatives (Specificity 45.0%)

False Positives = 22 | 1 = False Negatives

Accuracy = 62.3%, Precision = 47.6%, F1 = 63.5%, AU-ROC = 0.739

Coherently, the AU-ROC value of 0.739 falls inside the range defined by the series of 10-fold calculations in the previous section. All the presented CV calculations indicate that the SSIR models are stable and not spurious or fluctuating.

### 3.4. Virtual Generation of New Compounds and SSIR Predictions

Once the voting system is set up, it can be used to obtain predictions for proposals of new compounds. Within the SSIR approach, new virtual compounds can be easily defined, generating new combinations of residues along the substitution sites. In other words, new compounds are generated in silico simply by writing new strings of letters distinct from those presented in Table 3. It is only necessary to consider distinct combinations of the levels of Table 2. Then, each new candidate compound can be evaluated by the trained voting system in order to assess its potential activity.

#### 3.4.1. Restricted Generation of New Candidate Compounds

During the process of the virtual generation of new candidate compounds, the consideration of molecules that are far away from the molecular space defined by the training set must be avoided. It is necessary to rely on new structures that do not differ too much from those of training. This avoids producing extrapolations from the trained model. The new candidate molecules must be similar to the ones in training. A simple way to define similarity between two molecules in the SSIR context is simply by counting how many residues these molecules share in the same substitution sites. This is accomplished by comparing the molecular strings. For instance, the virtual compound ABC is more similar to the compound ABD than to the molecule BDD: In the first case, two residues are shared in the first and second sites, whereas in the second case, all residues in all sites are distinct. According to this simple approach, one can say that the index of similarity between ABC and ABD is 2, whereas it is zero when comparing ABC with BDD. The measure of proximity between two molecules can also be stated in terms of differences: the first pair differs by only one site, whereas the second pair differs by three sites.

Any new candidate can be compared individually with all the training molecules. This means that every new potential compound will get an index of similarity from each training compound. It is desirable that the candidate shows quite remarkable similarity indices (or a minimum number of site differences) with respect to a minimum number of training compounds. For a new candidate compound, having higher similarity indices with respect to the major number of training compounds indicates a higher proximity to the covered space by the expert voting system. In practice, this approximation to the training space is accomplished by accepting as new candidates only those differing by no more than a certain number of residues with respect to at least a minimum number of training compounds. As an example of the application of this rule of thumb, consider the database in Table 1 and the new virtual compound ADC. It can be checked that this virtual molecule differs by no more than one residue (similarity index of 2) with respect to a minimum of two training compounds. Specifically, ADC differs by only one residue with respect to the three compounds AAC, CDC, and DDC of Table 1. This condition ensures that the new proposed compound is quite close to the space spanned by the database. As another example, relative to the database of Table 1, there are only 19 new definable compounds (out of the 39 new definable in total) differing by no more than one substitution for at least two of the nine compounds of the database.

In particular, in this study, the new proposed virtual compounds must fulfill the following condition: They must have no more than two distinct residues (minimum similarity index of 7) with respect to three training compounds at least. This resulted in a new external set of 4760 compounds, which were selected from a pool of 2015939 new possible candidates. For instance, along the 61 training compounds, the string FABKBIDLJ (first candidate in Table 6) shows only one difference with respect to seven training items, two differences respect to 18, three differences with respect to 24, four differences with respect to five, five differences with respect to five, six differences with respect to one database item, and seven differences with respect to one training string.

#### 3.4.2. Predictions Among the Selected New Candidates

The predictions for the new set of virtual compounds have been obtained from the rules generated by the above case of multilevel rules of order 3 (with terms of order 2) with a *p*-value threshold of 0.15 (see Table 5). Each new compound was tested against 251,920 significant rules, and the assigned votes coming from this expert system were collected. In other words, the newly generated molecular set followed the test block of Figure 2, and it was treated as another test set, but this time without knowledge of the molecular activity. For each of the 4760 virtual molecules, the number of collected votes from the voting system ranged from a value of −4409 up to +7413 (for the training compounds, the range was from −4286 votes up to +7919 votes). This procedure generated a ranked list of molecules, and it is expected that the ones with more votes are the best candidates for exhibiting better anti-HIV activity. Table 6 shows the first 14 compounds leading the list and having more than 6500 votes. It is worth noting that the whole process of rule generation, voting system selection, and generation of new active molecular proposals is very fast. About only a few minutes are necessary for the whole process, and it can be done with a personal computer.

The chemical interpretation of the new molecules is mainly restricted to the interpretation of the frequencies of the chemical residues (letters in the strings) that appear in Table 6. If the list of candidates is limited to those of this table, it can be concluded that, at the first substitution site, an oxygen atom (level F of Table 2) is preferred. At the same time, residues C(R) and C(S) (carbon atoms in any absolute configuration or levels A and B) with a hydrogen atom completing the valence should be chosen as substitutions at site 2 (but not nitrogen). Additionally, at positions 3 and 4, a -CH_2_ (level B) and -CHαF (level K) must be fixed, respectively. At position 6, the presence of a hydrogen atom (level I) is necessary. Of course, only experimental synthesis and evaluations can reveal *a posteriori* if the proposed list of compounds is mostly of interest.

## 4. Conclusions

The SSIR with multilevel terms, a systematic procedure for ranking series of combinatorial analogues, has been presented in the context of using symbolic multilevel descriptors. Alongside the general description of the method, an illustrative application example has been provided. The approach has proven to be straightforward to implement, fast, and systematic. Cross-validation procedures have also been conducted, showing the model’s stability. It has been shown that the incorporation of multilevel descriptors contributes to improved performance with respect to the former classical SSIR procedure. At the same time, it has been shown how the procedure can compete with another well-established classification methodology. Finally, it has been shown how the generation of new molecular proposals is systematic and flexible.

## Figures and Tables

**Table 1:** Molecular substitution codification for the compound family example. Each letter and its position represent a distinct chemical residue placed at the corresponding substitution site.

**Compound**	AAA	AAB	AAC	BCC	BDA	CDC	CAA	DBB	DDC
**Of interest?**	Y	Y	Y	N	N	N	N	N	N

**Table 2:** Residue codifications of the compounds of Table 3 in reference [6]. Note that some pairs of sites in the original publication here have been coded in a fused form.

Site (Available Residues)	Sites in Original Publication	Level	Residue
1	V R_1_	A	CHCH_2_OH
(8)		B	C=CH_2_
		C	CH_2_
		D	CHαF
		E	CHβF
		F	O
		G	OCH_2_
		H	S
2	W R_2_	A	C(R)
(3)		B	C(S)
		C	N(S)
3	X R_3_	A	=CH
(5)		B	CH_2_
		C	CHF
		D	CHOH
		E	O
4	Y R_4_	A	C=NOCH_3_
(22)		B	C=NOCOCH_3_
		C	C=NOH
		D	CCF3
		E	CH
		F	CH_2_
		G	CHαCF_3_
		H	CHαCH_2_OH
		I	CHαCN
		J	CHαC≡CH
		K	CHαF
		L	CHαN_3_
		M	CHαNH_2_
		N	CHαNO_2_
		O	CHαOCH_2_-
		P	CHαOH
		Q	CHαSCOCH_3_
		R	CHβN_3_
		S	CHβN(OH)CH_2_Ph
		T	O
		U	OH
		V	S
5	Z	A	C(R)
(2)		B	C(S)
6	R_5_	A	C_2_H_5_
(12)		B	CF_3_
		C	CH_2_N_3_
		D	CH_3_
		E	CH=CH_2_
		F	CH=CHCl
		G	CN
		H	C≡CH
		I	H
		J	N_3_
		K	NH_2_
		L	O
7	R_6_	A	CH(OH)CH_2_OH
(5)		B	CH(OH)CH_3_
		C	CH_2_CH_2_OH
		D	CH_2_OH
		E	H
8	R_7_	A	NH(CH_2_)_3_CH_3_
(15)		B	NH(CH_2_)_3_COOCH_3_
		C	NH(CH_2_)_3_COOH
		D	NH(CH_2_)_3_NH_2_
		E	NH_2_
		F	NHCH_2_CH=CH_2_
		G	NHCH_2_C≡CH
		H	NHCH_3_
		I	NHNH_2_
		J	NHOH
		K	OCH_3_
		L	OH
		M	SCH_3_
		N	SH
		O	Imidazole
9	R_8_	A	Br
(15)		B	C_2_H_5_
		C	CF_3_
		D	CH_2_CONH(CH_2_)_10_NH_2_
		E	CH_2_CONH(CH_2_)_12_NH_2_
		F	CH_2_CONH(CH_2_)_6_NH_2_
		G	CH_2_CONH(CH_2_)_8_NH_2_
		H	CH_2_NH(CH_2_)_6_NHCOCF_3_
		I	CH_2_NHCH_2_C≡CH
		J	CH_3_
		K	CH=CHBr
		L	Cl
		M	F
		N	H
		O	I

**Table 3:** Molecular family of Estrada’s reference codified in SSIR mode according to the equivalences stated in Table 2 above. The original compound No. 131 has been removed. Labels a and b stand for Ex1 and Ex2 external prediction sets, respectively. The remaining molecules belong to the TRN set. See text for details.

No.	SSIR Codification	Active? ^1^	Original Number in Ref. [6] ^2^
1	FABFBIDLJ	+1	1
2	FABFBIDEN	+1	2
3	FABFBIDLN	−1	3
4	FABFBIDLB	−1	4
5	FABFBIDAN	−1	5a
6	FABFBIDBN	−1	6
7	FABFBIDCN	−1	7
8	FABFBIDDN	−1	8
9	FABLBIDLN	+1	9
10	FABLBIDLJ	+1	10
11	FABRBIDLJ	−1	11
12	FABLBIDLB	−1	12a
13	FABLBIDLL	+1	13
14	FABLBIDEN	+1	14a
15	FABLBIDEJ	+1	15
16	FABLBIDHJ	−1	16
17	FABLBIDMJ	−1	17a
18	FABNBIDLJ	+1	18a
19	FABKAIDLN	+1	19
20	FABKAIDLJ	+1	20
21	FABKAIDNJ	+1	21
22	FABKAIDLL	+1	22
23	FABKAIDLA	+1	23
24	FABKAIDLO	+1	24
25	FACKAIDEJ	+1	25
26	FACFBIDLJ	−1	26
27	FABFBIDEJ	−1	27a
28	FABKAIDLB	−1	28
29	FABKAIDLM	−1	29
30	FABKAIDMN	−1	30
31	FABKAIDKN	−1	31
32	FABMBIDHJ	−1	32a
33	FABGBIDLJ	−1	33a
34	FABGBIDLN	−1	34a
35	HABHBIDLK	−1	35a
36	FABFBIDLL	−1	36a
37	FADIBIDLJ	−1	37a
38	FADJBIDLJ	−1	38
39	FABMBIDMJ	−1	39
40	CBEFBJDEN	−1	40
41	CBEFBKDEN	−1	41a
42	CABTACDLJ	−1	42
43	CABTACDLN	−1	43
44	FABLAICLJ	−1	44
45	FABLBIALJ	−1	45
46	FABLBIBLJ	−1	46a
47	FABLAIALJ	−1	47
48	FABLAIBLJ	−1	48a
49	FABCBIDLJ	+1	49
50	FABABIDLJ	−1	50
51	FABBBIDLJ	+1	51
52	FAAEBIDLJ	+1	52a
53	FAAEBIDEN	+1	53
54	FAAEBIDEL	−1	54
55	FAADBIDLJ	−1	55
56	FAAEBIDLN	−1	56a
57	FAAEBIDLL	−1	57a
58	FABVBIDEN	+1	58
59	FABVBIDIN	−1	59a
60	FABVBIDON	−1	60
61	FABTAIDEJ	−1	61
62	FABTAIDEN	−1	62
63	FABTAIDLN	−1	63
64	FABTAIDLM	−1	64a
65	FABTAIDLL	−1	65
66	FABTAIDLA	−1	66
67	FABTAIDLO	−1	67a
68	FABTAIDLB	−1	68
69	FABTAIDLC	−1	69
70	FABTAIDEM	−1	70a
71	FABTAIDEA	−1	71a
72	FABTAIDEO	−1	72a
73	HABTAIDLJ	−1	73a
74	HABTAIDLN	−1	74a
75	FABVBIDJN	−1	75
76	FABVBIDAN	−1	76
77	FABVBIDHN	−1	77
78	FABVBIDGN	−1	78
79	FABVBIDFN	−1	79
80	FABVBIDEM	+1	80a
81	HABTAIDEM	+1	81
82	FABPAGDEN	+1	82a
83	FABPAEDLJ	+1	83a
84	FABPAEDEN	+1	84a
85	FABPAFDEN	+1	85a
86	FABPAHDEN	+1	86a
87	FABPADDEN	+1	87a
88	FABPAADEN	+1	88
89	CCBFAIDLN	−1	89a
90	CCBFAIDLJ	−1	90a
91	CCBFAIDEN	−1	91a
92	GBBPAIDLN	−1	92a
93	GBBPAIDLM	−1	93a
94	GBBPAIDLO	−1	94a
95	GBBPAIDLB	−1	95a
96	GBBPAIDLK	−1	96a
97	GBBPAIDEM	−1	97a
98	GBBPAIDEL	−1	98
99	GBBPAIDEO	−1	99a
100	FBBLBIDMJ	−1	100
101	FBBKAIDMN	−1	101a
102	FBBVAIDEN	+1	102
103	HBBTBIDLJ	−1	103a
104	HBBTBIDLN	−1	104a
105	FBBVAIDEM	+1	105a
106	HBBTBIDEN	+1	106a
107	HBBTBIDEM	+1	107
108	HBBTAIDLJ	−1	108
109	HBBTAIDLN	−1	109a
110	HABTBIDLJ	−1	110a
111	HABTBIDLN	−1	111a
112	FBAEAIDLF	−1	112a
113	FBAEAIDLG	−1	113a
114	FBAEAIDLD	−1	114a
115	FBAEAIDLE	−1	115a
116	DBBKAIDLJ	−1	116a
117	FABOALDLJ	−1	117
118	FABPAHDLJ	+1	118b
119	FABQBIDLJ	−1	119b
120	FABPAJDLJ	+1	120b
121	FABSBIDLJ	−1	121b
122	FABMBIDLJ	−1	122b
123	FABIBIDLJ	−1	123b
124	FAAEBIDLH	−1	124b
125	FAAEBIDLI	−1	125b
126	FAAEBBDLJ	−1	126b
127	BABUAIDLN	−1	127b
128	BABUAIDEN	−1	128b
129	ABBFAIELL	−1	129b
130	ABBFAIELJ	−1	130b
131	EBBLBIDLJ	−1	132b
132	FABFAIDLJ	−1	133b

^1^ Molecular activity (+1: active or compound of interest presenting an EC_50_ ≤ 10 µM on MT4 assay; −1: not active or compound of non-interest because it presents an EC_50_ > 10 µM). ^2^ Numerical codification in the original publication (note that compound 131 is skipped). Labels a and b stand for external sets. See the text for details.

**Table 4:** Training and test AU-ROC values when classical SSIR rules are being considered (negation terms have not been considered). The molecular set of training determines the total number of rules to be analyzed and statistically evaluated. AU-ROC for external Ex1 and Ex2 sets is reported. See text for more details.

Rules Order ^a^	*p*-Value Threshold (%)	AU-ROC_TRN_ ^b^	AU-ROC_Ex1_	AU-ROC_Ex2_
1 (56)	1	-	-	-
	3	0.652 [1] 0.602	0.784	0.462
	5	0.750 [2] 0.602	0.761	0.462
	10	0.750 [2] 0.702	0.761	0.462
	15	0.764 [3] 0.683	0.788	0.462
	20	0.764 [3] 0.713	0.788	0.462
2 (1398)	1	-	-	-
	3	0.713 [6] 0.535	0.814	0.462
	5	0.821 [17] 0.618	0.848	0.462
	10	0.848 [20] 0.763	0.830	0.423
	15	0.864 [27] 0.744	0.839	0.423
	20	0.874 [30] 0.736	0.804	0.115
3 (18376)	1	-	-	-
	3	0.728 [10] 0.486	0.844	0.462
	5	0.861 [54] 0.561	0.842	0.423
	10	0.857 [68] 0.727	0.832	0.423
	15	0.909 [96] 0.772	0.853	0.269
	20	0.925 [106] 0.639	0.859	0.423
4 (145573)	1	-	-	-
	3	0.714 [7] 0.369	0.852	0.500
	5	0.874 [87] 0.490	0.841	0.423
	10	0.886 [119] 0.615	0.838	0.423
	15	0.933 [188] 0.723	0.843	0.423
	20	0.934 [200] 0.661	0.848	0.462

^a^ Rule order and total number of rules definable. ^b^ AU-ROC value for training (AU-ROC_TRN_), number of significant rules found (in squared brackets) forming the voting system (i.e., selected by its *p*-value), and AU-ROC value for the process of leave-one-out CV among the training items, AU-ROC_L1O_. See text for details.

**Table 5:** Training and test AU-ROC values when multilevel SSIR models involving up to terms of order 2 are considered. The molecular set of training determines the total number of definable rules. See text for more details.

Rules Order ^a^	*p*-Value Threshold (%)	AU-ROC_TRN_ ^b^	AU-ROC_Ex1_	AU-ROC_Ex2_
1 (311)	1	0.752 [2]	0.798	0.462
	3	0.837 [17]	0.833	0.769
	5	0.833 [19]	0.832	0.769
	10	0.830 [34]	0.837	0.769
	15	0.843 [39]	0.833	0.769
	20	0.883 [53]	0.854	0.500
2 (34703)	1	0.927 [61]	0.828	0.231
	3	0.912 [454]	0.825	0.308
	5	0.908 [749]	0.852	0.846
	10	0.876 [1137]	0.864	0.846
	15	0.893 [1757]	0.842	0.846
	20	0.919 [2194]	0.853	0.846
3 (1745977)	1	0.961 [595]	0.829	0.231
	3	0.945 [4524]	0.853	0.192
	5	0.924 [9935]	0.874	0.846
	10	0.894 [14663]	0.865	0.846
	15	0.936 [29608]	0.863	0.846
	20	0.955 [35232]	0.857	0.769
4 (44701344)	1	0.956 [2773]	0.852	0.423
	3	0.954 [21967]	0.874	0.308
	5	0.945 [63193]	0.878	0.769
	10	0.932 [94530]	0.887	0.731
	15	0.967 [251920]	0.865	0.692
	20	0.968 [284850]	0.876	0.462

^a^ Rule order and total number of rules generated and evaluated. ^b^ AU-ROC value for training and number of significant rules found (in squared brackets) according to the selected *p*-value threshold.

**Table 6:** First-ranked newly proposed compounds according to the assigned votes of the significant rules. Each compound differs by no more than two letters from at least three training compounds. See text for details.

Rank Position	SSIR Codification	Votes
1	FABKBIDLJ	7413
2	FBBKAIDLJ	7385
3	FABKBIDLO	7080
4	FABKBIDLL	7010
5	FBBKAIDLO	6992
6	FABKAIDEJ	6979
7	FBBKAIDLL	6944
8	FBBKBIDLJ	6891
9	FABKAICLJ	6850
10	FABKAIALJ	6838
11	FABKBIDEJ	6830
12	FABKBIDLA	6689
13	FBBKAIDLA	6650
14	FABKAIDEN	6520

## Data Availability

The raw data supporting the conclusions of this article will be made available by the authors on request.

## Abbreviations

The following abbreviations are used in this manuscript:

SSIR	Superposing Significant Interaction Rules;
HIV	Human immunodeficiency virus;
TOPS-MODE	Topological sub-structural molecular design;
AU-ROC	Area under receiver operating characteristic;
TRN	Training;
CV	Cross-validation;
L1O	Leave-one-out;
L2O	Leave-two-out;
BL2O	Balanced leave-two-out;
EC_50_	Effective concentration reaching 50% of maximum effect.
